# Amazon drought resilience – emerging results point to new empirical needs

**DOI:** 10.1111/nph.18670

**Published:** 2023-01-05

**Authors:** Rutuja Chitra‐Tarak, Jeffrey M. Warren

**Affiliations:** ^1^ Earth and Environmental Sciences Division Los Alamos National Laboratory Los Alamos NM 87545‐1663 USA; ^2^ Environmental Sciences Division Oak Ridge National Laboratory Oak Ridge TN 37831 USA

**Keywords:** Amazon, climate change forecasting, drought tolerance, dynamic global vegetation models, ecohydrology, hydrological niches, land surface models, tropical forests

## Abstract

This article is a Commentary on Costa *et al.*, **237**: 714–733.

Will climate change turn tropical forests from a carbon sink to a source (Pan *et al*., 2011)? Tropical forests cycle more carbon, water, and energy than any other biome (Bonan, 2008). The Amazon is the largest tropical forest, spanning one‐third of South America, and any perturbation in its function has repercussions on the global climate. Mild and severe droughts in the Amazon are predicted to double and triple in area, respectively, by 2100, while the area under wet extremes will increase (Duffy *et al*., 2015). Our understanding of the role of plants' hydrological environments in determining their drought response, however, is limited (Chitra‐Tarak *et al*., 2018, 2021). An important review by Costa *et al*. (2023; pp. 714–733) published in this issue of *New Phytologist* highlights that shallow‐water table (WT) forests constitute *c*. 50% of the Amazon and may act as hydrological refugia during droughts, yet they have been neglected in Amazon forest research to date. Emerging results suggest that the shallow‐WT forests that dominate the Amazon basin may increase in productivity under moderate droughts due to relief from hypoxia, suggesting a potential buffer from drought relative to deeper WT forests (Fig. 1). During severe droughts, however, shallow‐WT forests may be vulnerable to collapse due to drought‐intolerant traits. Addressing the underrepresentation of relatively drought‐resilient shallow‐WT forests in the Amazon's forest inventories may help resolve the much‐debated incongruence in recent studies of the Amazon's drought resilience. Data from forest inventories (biased toward deep‐WT forests) indicate that the Amazon's carbon sink is declining, partially because of drought impacts (Phillips *et al*., 2009; Brienen *et al*., 2015), whereas basin‐wide satellite‐based measures of gross primary productivity, which include shallow‐WT forests, indicate varied regional responses to droughts (Saleska *et al*., 2007; Brando *et al*., 2010). Costa *et al*.'s (2023) descriptive insights of the intricate balance of geology, topography, hydrology, vegetation, and drought on ecosystem function, as well as their conceptual predictive framework, are useful for developing new empirical research in these understudied ecosystems and improving Earth system models.

**Fig. 1:**
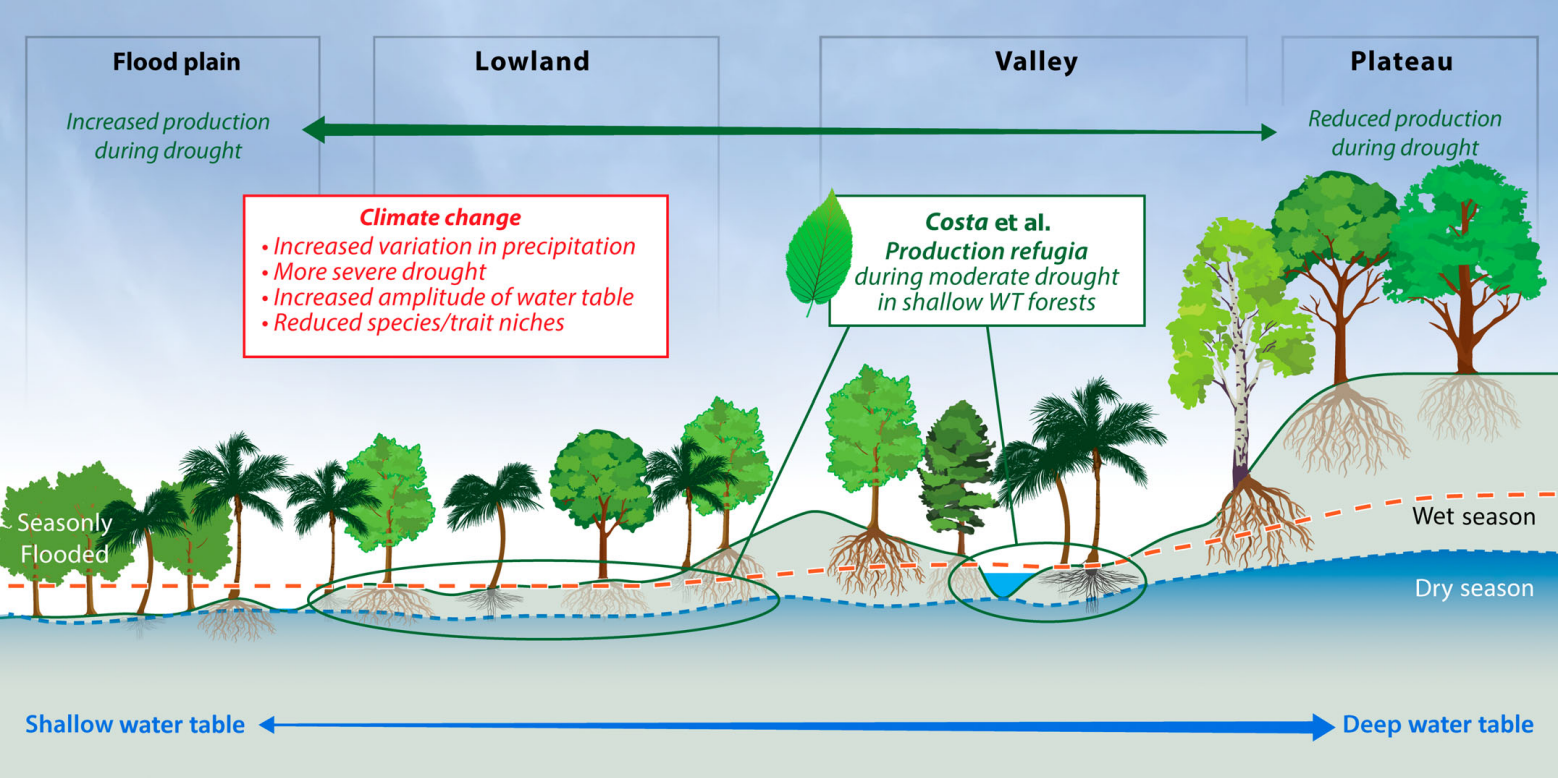
Costa *et al*. (2023; pp. 714–733) highlight in this issue of *New Phytologist* the hydrodynamic trait variation in rooting depth, xylem anatomy, phenology, and hydraulic strategy in response to gradients in water‐table depth. This variation may lead to enhanced production in lowland forests during moderate drought, offsetting reduced production in uplands, yet extreme drought conditions could drop the water table below the rooting zone of the mesic lowland forests leading to catastrophic loss and tipping point for the Amazon. Image credit: ORNL Creative Services.

Focusing on mathematical relationships among plant traits, performance, environmental gradients, and environmental change is likely to provide the predictive ecological principles that Earth system models need (McGill *et al*., 2006). Costa *et al*. (2023) outlined the scale of diversity in hydrological environments of the Amazon, highlighting the underrepresentation of shallow‐WT forests in forest research networks. They focused on the key differences in hydrological regimes among shallow‐WT forests, the distinctive traits that shape their structure and dynamics, and the emerging results, assessing the impact of droughts on growth and mortality rates of shallow‐WT forests from recent hydrologically designed plots in the central Amazon. Finally, they provided hypotheses regarding basin‐wide responses for shallow‐ vs deep‐WT forests under intensifying droughts and suggest a research agenda to rapidly fill the knowledge gaps for shallow‐WT forests.

‘The underrepresentation of shallow‐WT forests in ecological studies highlights a critical gap in understanding their sensitivity to drought such that any model projections of these ecosystems cannot be validated and projections will likely be biased toward deep‐WT ecosystems.’

Costa *et al*. (2023) described hydrological patterns in the Amazon that are normally confined to hydrological literature, and how the hydrological regime of shallow‐WT forests modulates traits, structure, and dynamics. Costa *et al.*'s (2023) reanalysis of ground WT depth products places 50% of Amazon area under shallow‐WT conditions (≤ 5 m), an additional 37% with medium‐WT conditions (5–20 m), 8% with deep‐WT conditions (> 20 m), and 5% under seasonally flooded forests. Young geologies in the Amazon basin have flatter landscapes, where shallow‐WT locations have high WT variation coupled to precipitation, whereas old geologies have rolling and deeply incised terrains, where WT depth follows topography due to lateral drainage from plateaus to valleys. Here, shallow WTs are restricted to the margins of streams and valleys, which have low WT variation buffered by head storage in the plateaus, with WT peaking at the beginning of the dry season and varying with climatic seasonality. Seasonal or aseasonal waterlogging in shallow‐WT forests creates alternating favorable and unfavorable growth conditions. Costa *et al*. (2023) reviewed the mechanisms by which hypoxic conditions structure shallow‐WT forests: hypoxic environments prevent deep rooting and reduce root permeability, stomatal conductance, and nutrient availability. Roots under these conditions switch to alcoholic fermentation that reduces energy yield from respiration (from 36 to 2 ATP per glucose molecule). As a result, despite their acquisitive traits, such as, higher specific leaf area and xylem vessel diameter, shallow‐WT forests have shorter, smaller diameter trees, lower productivity, and lower total stand biomass (but not in dry climates). Reduced root anchorage, lower wood density, and higher vulnerability to embolism of shallow‐WT forests are associated with higher mortality rates and high biomass turnover.

Costa *et al*. (2023) considered an intermediary drought regime in which productivity is maximized in the shallow‐WT topographic positions. This hydrological zone allows moderate soil drying and development of a deeper, more aerobic soil zone above the WT that enhances growth rates while minimizing mortality. Given higher embolism vulnerability of shallow‐WT forests, Costa *et al*. (2023) suggested that these forests may be particularly likely to succumb to severe drought. Plant physiological tolerances, functional traits, and performance (growth, survival, and reproduction) are optimized over an environment due to trait trade‐offs such that performance decreases away from the optima. Costa *et al*. (2023) thus expected that the whole forest functional response will be in proportion to the perturbation in the plants' historical hydrological regime, such as, the frequency distribution of soil water variation.

Some evidence supports that drought strategies are shaped by species‐specific hydrological niches – the historical moisture regime (distribution) along trees' rooting profile vertically belowground through soil, rock, or WT, a function of species' habitat associated with respect to topography, climate, and soil (Chitra‐Tarak *et al*., 2018). A safety‐efficiency trait trade‐off may underlie hydrological niche segregation: tree species associated with a hydrological niche of fluctuating soil moisture (e.g. shallow‐rooted species) are found to be less vulnerable to embolism at low water stress (safety) than those associated with a reliable, stable water source (e.g. deep‐rooted species), which have greater hydraulic conductivity (efficiency) but are more vulnerable to embolism at low water stress (Chitra‐Tarak *et al*., 2021). Evidence also supports that the extent of perturbation relative to a species' hydrological niche matters in eliciting a drought‐induced mortality response. Despite their higher embolism vulnerability, deep‐rooted tree species were found to survive several El‐Niño droughts over 35 yr in a moist tropical forest compared with shallow‐rooted species, because annual recharge of deep‐water reserves limited drought exposure for these deep‐rooted species (Chitra‐Tarak *et al*., 2021). More extreme or prolonged drought may expose deep‐rooted trees to water stress greater mortality rates relative to shallow‐rooted species, as was found in a dry tropical forest (Chitra‐Tarak *et al*., 2018).

Costa *et al*. (2023) focused on the prominent trend of mean‐community rooting depths and traits along spatial water‐table gradients, but within‐community trait diversity, including drought avoidance via leaf loss, can be a large contribution to drought resilience in tropical forests (Fan *et al*., 2017; Chitra‐Tarak *et al*., 2018; Oliveira *et al*., 2019). At high topography locations with deep WTs, shallow‐rooted and deep‐rooted tree species (and sizes) may co‐exist on distinct vertical and temporal hydrological niches and experience contrasting moisture dynamics, the former associated with a high amplitude of variation in shallow soil moisture *coupled* to precipitation and the latter associated with high soil moisture at depth *decoupled* from precipitation (Chitra‐Tarak *et al*., 2018, 2021). The decoupling of WT dynamics from precipitation is a memory effect generated by long vertical travel times for water and declining water extraction by depth. This general phenomenon occurs at seasonal, multi‐annual, and decadal scales depending on climate, seasonality, and weathered zone depth, and is enhanced by drought duration and intensity (Ruiz *et al*., 2010; Ivanov *et al*., 2012). How shifting precipitation patterns in the Amazon and beyond will perturb the hydrological regimes and narrow spatio‐temporal niches remains to be investigated.

Land surface models are vital tools to understand and predict the impact of vegetation–hydrology interactions and other surficial dynamics, on the Earth system under global climate change (Fisher & Koven, 2020). Land surface models host dynamic global vegetation models (DGVMs). Although most DGVMs currently use a big leaf approach, often representing tropical forests with a single plant functional type, next‐generation DGVMs, the cohort‐based vegetation demographic models (VDMs), efficiently represent functional and structural diversity of forests at regional to global scales (Fisher *et al*., 2018). In VDMs, vegetation structure and distribution emerge from the first principles of community and physiological ecology: plant functional traits interacting with the environment under competition and disturbance. Vegetation demographic models can represent a diversity of microenvironments by decomposing a landscape into patches of similar ages since disturbance. Currently, however, most VDMs share the same soil water pool across patches, underestimating the heterogeneity in plant water environments and its feedback on community composition and surface energy balance (Fisher *et al*., 2018). The host land surface models need to better represent landscape heterogeneity at the spatial scale they are typically deployed at 0.5°–2° to better capture lateral flow from hills to valleys and variation due to slope aspect (sunny vs shady slope), the two first‐order controls on water, and energy availability across the landscape (Fan *et al*., 2019). Model developments are limited by the availability of landscape‐scale concurrent observations for hydrological stores and fluxes (e.g. water‐table depths, soil moisture, evaporation, transpiration, discharge), parameters for soil water retention curves and hydraulic conductivity at depth, and community‐scale rooting depths and their temporal dynamics, leaf phenology, trait covariation with other hydraulic traits, and phenotypic plasticity. Models also need to improve the representation of interactions among hypoxia, nutrient uptake, and root dynamics.

Although Costa *et al*. (2023) focused on shallow‐WT forests that are not seasonally inundated, they acknowledged that their analysis might be extended to floodplain forests as an endmember. Even, small watersheds with ephemeral streams along local valley floors may periodically flood. Such floodplain forests occur in lowlands pantropically, including the Amazon, the expansive Pantanal south of the Amazon, the Kakadu region in northern Australia, and the Tonle Sap/Mekong River floodplain (Parolin *et al*., 2016).

The underrepresentation of shallow‐WT forests in ecological studies highlights a critical gap in understanding their sensitivity to drought such that any model projections of these ecosystems cannot be validated and projections will likely be biased toward deep‐WT ecosystems. Costa *et al.*'s (2023) timely, well‐written, and detailed review holds broad interest for empiricists and modelers of plant ecohydrology, physiology, community ecology, biogeography, tropical forests, and climate change ecology.
